# Positive Selection and Functional Divergence of *R2R3-MYB* Paralogous Genes Expressed in Inflorescence Buds of *Scutellaria* Species (Labiatae)

**DOI:** 10.3390/ijms16035900

**Published:** 2015-03-13

**Authors:** Bing-Hong Huang, Erli Pang, Yi-Wen Chen, Huifen Cao, Yu Ruan, Pei-Chun Liao

**Affiliations:** 1 Department of Life Science, National Taiwan Normal University, 88, Ting-Chow Rd., Sec. 4, Taipei 116, Taiwan; E-Mail: 80243011s@ntnu.edu.tw; 2 Laboratory of Computational Molecular Biology, College of Life Sciences, Beijing Normal University, Beijing 100875, China; E-Mails: pangerli@bnu.edu.cn (E.P.); hfcao@mail.bnu.edu.cn (H.C.); 3 Department of Biological Science and Technology, National Pingtung University of Science and Technology, 1, Shuefu Rd., Neipu, Pingtung 912, Taiwan; E-Mail: m10118029@mail.npust.edu.tw; 4 School of Life Science and Engineering, Chongqing Three Gorges University, Chongqing 404001, China; E-Mail: ruanyu2@163.com; 5 The College of Forestry, Beijing Forestry University, Beijing 100083, China

**Keywords:** anthocyanin, evolutionary rate, functional divergence, phylogenetic analysis, positive selection, *R2R3-MYB*, *Scutellaria*

## Abstract

Anthocyanin is the main pigment forming floral diversity. Several transcription factors that regulate the expression of anthocyanin biosynthetic genes belong to the *R2R3-MYB* family. Here we examined the transcriptomes of inflorescence buds of *Scutellaria* species (*skullcaps*), identified the expression *R2R3-MYBs*, and detected the genetic signatures of positive selection for adaptive divergence across the rapidly evolving skullcaps. In the inflorescence buds, seven *R2R3-MYBs* were identified. *MYB11* and *MYB16* were detected to be positively selected. The signature of positive selection on *MYB* genes indicated that species diversification could be affected by transcriptional regulation, rather than at the translational level. When comparing among the background lineages of *Arabidopsis*, tomato, rice, and *Amborella*, heterogeneous evolutionary rates were detected among *MYB* paralogs, especially between *MYB13* and *MYB19*. Significantly different evolutionary rates were also evidenced by type-I functional divergence between *MYB13* and *MYB19*, and the accelerated evolutionary rates in *MYB19*, implied the acquisition of novel functions. Another paralogous pair, *MYB2*/*7* and *MYB11*, revealed significant radical amino acid changes, indicating divergence in the regulation of different anthocyanin-biosynthetic enzymes. Our findings not only showed that *Scutellaria R2R3-MYBs* are functionally divergent and positively selected, but also indicated the adaptive relevance of regulatory genes in floral diversification.

## 1. Introduction

Plant diversity is usually characterized by morphological variation [1,2]. Adaptive divergence is one of the most important mechanisms for the high divergence of morphological traits among phylogenetically closely related species [3]. Based on population genomic analyses, both functional genes and regulatory elements have been found to be associated with the variation of adaptive traits [4]. Genes encoding transcription factors (TFs) are evidenced to be relevant to rapid speciation [5,6,7]. In this study, we identified the signatures of positive selection and functional divergence of duplicated TFs in the rapidly divergent herb genus *Scutellaria* (Labiatae), commonly known as skullcaps.

There are eight *Scutellaria* species on the continental island of Taiwan, and six are endemic species. All Taiwanese *Scutellaria* have the same chromosome numbers (2*n* = 26) [8]. Taiwan Island is rugged in topography, with a land area of approximately 36,000 km^2^ and located near the southeastern mainland of China. These Taiwanese skullcaps have been suggested to have quickly evolved and recently speciated via recurrent dispersal and geographic isolation events during the Pliocene and Pleistocene [9]. Divergent growth habitats with small population sizes of Taiwanese endemic skullcaps accelerated the fixation rate of alleles under synchronized selective pressure and stochastic drift, as is represented by the fixed haplotypes of chalcone synthase (CHS) and cinnamyl alcohol dehydrogenase (CAD) in several populations of *Scutellaria taiwanensis*, *Scutellaria tashiroi*, *Scutellaria playfairii*, *Scutellaria austrotaiwanensis*, and *Scutellaria indica* in Taiwan [9]. CHS is an upstream regulatory enzyme of anthocyanin biosynthesis that catalyzes the conversion of 4-coumaroyl-CoA and malonyl-CoA to naringenin chalcone and interacts with other anthocyanin biosynthesis enzymes, including chalcone isomerase (CHI), flavanone 3-hydroxylase (FSH), dihydroflavonol 4-reductase (DFR), flavanone 3-hydroxylase (F3H), *etc.* [1,10]. The variable expression of these anthocyanin biosynthesis enzymes between species corresponds with the diversity of flower-color types [1], and they are regulated by several *R2R3-MYB* TFs [11,12].

*R2R3-MYBs* function in the control and regulation of secondary metabolism in plants [13], and they also play a role in the development of the axillary meristems of inflorescences [14,15], influencing flower morphogenesis [16,17], and color [18,19,20,21,22]. Certain *R2R3-MYBs* that are expressed in flowers differentially regulate the anthocyanin biosynthetic enzymes and cause flower-color divergence, which has resulted in the incipient speciation between two parapatric ecotypes of *Mimulus aurantiacus* [19] and between the sympatric, phylogenetically closely related species *Phlox drummondii* and *Phlox cuspidata* via differentiating the pollinator preference [23]. Among these speciation cases, divergent selection plays a key role in determining the regulation level of *R2R3-MYBs* on pigment accumulation. Taiwanese skullcaps vary in growth forms, microtraits (e.g., pollen size and exine), and variegation in flower petals (Table 1). Although the Taiwanese *Scutellaria* species have only few genetic variations between species, the clear and distinguishable morphological characters support that these species belong to different taxonomic units. Multilocus markers (the microsatellite DNA and amplified fragment length polymorphism loci) also suggest that the Taiwanese *Scutellaria* species are recently and rapidly divergent species [24]. For exploring the genetic mechanism on the rapid divergence of these island endemic herbs, we reconstructed the transcriptomic library of inflorescence buds of four Taiwanese *Scutellaria* species, including the widespread species *S. indica* and three endemic species *S. tashiroi*, *S. playfairii*, and *S. taiwanensis*, to determine putative genes functioning to control flower morphogenesis and color, and thereby permit exploration of the evolutionary genetic mechanisms underlying the rapid speciation of the *Scutellaria*.

*R2R3-MYBs* are characterized by the *MYB* domain, which is responsible for the regulatory specificity of corresponding proteins, and the highly variable *C*-terminal region, which is less important for the regulatory function [25,26]. A recent study has identified 19 *MYBs* in *Scutellaria baicalensis*, of which 11 *MYBs* contain conserved *R2R3* domains and motifs [27]. The gene ontology (GO) annotation suggests that these *SbMYBs* function in the response to plant hormone and environmental stresses, and regulation of the circadian rhythm, flowering periods, and pigment biosynthesis [27]. Physiological experiments in *S. baicalensis* and other model species have provided further evidence for their functions in regulating anthocyanin biosynthesis and pigment accumulation, stress tolerance, and meristems and flowers development [12,17,27,28,29,30,31,32,33] (Table 2). In the transcriptomic database of inflorescence buds of the four Taiwanese *Scutellaria* species *S. indica*, *S. tashiroi*, *S. playfairii*, and *S. taiwanensis*, we found large amount of reads belongs to the R2R3-MYB transcription factors. This indicates high expression of *R2R3-MYBs* in inflorescence buds of *Scutellaria* and may imply that these genes are important in the regulation and/or adaptation to the environments [34].

**Table 1:** Pollen type and growth form of four Taiwanese *Scutellaria* species used in this study.

Species	Pollen		Growth Form		
	Size (μm)	Exine	Stem	Inflorescence	Petal and Corolla
*S. indica*	(18–23) × (12–17)	Finely reticulate	Erect or procumbent at base	Terminal loose raceme	Geniculate at base, purple, pink or white
*S. tashiroi*	(18–22) × (14–17)	Loose reticulate to rugulate	Slender, procumbent, tufted	Axillary, seldom terminal raceme	Curve at base, dark purple
*S. playfairii*	(16–20) × (10–15)	Loose reticulate to rugulate	Erect, seldom tufted	Terminal loose raceme	Geniculate at base, whitish purple
*S. taiwanensis*	(23–30) × (16–20)	Irregular rugulate	Erect, often tufted	Terminal loose raceme	Geniculate at base, white with purple spot

**Table 2:** Putative functions of *Scutellaria* R2R3-MYBs.

Group	Function	Species	Reference
*MYB2/7/11*	Shoot and axillary meristems formation	*Arabidopsis thaliana* (S14) ^a^	[28]
	Flavonoid regulation through GA metabolism; regulate *PAL*, *C4H*, *CHS*, *CHI* and *UFGT*	*Scutellaria baicalensis*	[27]
*MYB8*	Induction of anthocyanin accumulation	*Arabidopsis thaliana* (S6) ^a^ and *Nicotiana tabacum*	[29]
	Sharing similar expression pattern with *C4H* and *CHS* after GA treatment	*Scutellaria baicalensis*	[27]
*MYB13/19*	Alternation of expression level of anthocyanin biosynthesis genes and pigment accumulation under cold stress	*Arabidopsis thaliana* (*AtMYB3*)	[12]
		*Brassica oleracea*	[30]
		*Nicotiana tabacum*	[31]
*MYB15*	Regulation of serine/threonine protein phosphatases to enhance salt or drought tolerance	*Arabidopsis thaliana* (*AtMYB20*)	[32,33]
*MYB16*	MicroRNA regulation and anther and pollen development	*Arabidopsis thaliana* (S18) ^a^	[17]

^a^ Subgroup of *MYB* family defined by Kranz *et al.* [28]. S14: *AtMYB68*, *AtMYB36*; S6: *AtMYB90*; S18: *AtMYB65*; GA: Gibberellic acid; PAL: Phenylalanine ammonia lyase; C4H: Cinnamate 4-hydroxylase; CHS: Chalcone synthase; CHI: Chalcone isomerase; UFGT: UDP-glucose flavonoid glucosyl-transferase.

To examine the evolutionary role of the *R2R3-MYBs* for the adaptive divergence of skullcaps, we identified the *R2R3-MYBs* from transcriptomes extracted from the inflorescence buds of four phylogenetically closely related species, *S. taiwanensis*, *S. indica*, *S. playfairii*, and *S. tashiroi*, and collected sequences from the published shoot transcriptome of *Scutellaria montana* from the online 1KP database (Accession ID: ATYL) and *R2R3-MYBs* of *S. baicalensis* from NCBI GenBank. Four specific questions were addressed here: (1) What kind of *R2R3-MYBs* are expressed in the inflorescence buds? (2) Does selective pressure shape the evolution of the *R2R3-MYBs* in rapidly evolving skullcaps? (3) Are the recently duplicated paralogs of *R2R3-MYB* genes functionally divergent in skullcaps? (4) What are the evolutionary mechanisms of these recently duplicated genes? Based on data mining from the transcriptomes database, we collected *Scutellaria R2R3-MYB* genes expressed in the inflorescence buds and conducted genetic analyses to answer the above questions.

## 2. Results

### 2.1. Gene Annotation by Basic Local Alignment Search Tool (BLAST) Analyses

In our ~140-megabase transcriptomic libraries, the mean size of scaffolds is 630 bps and 76,750, 69,811, 44,544, and 69,921 unique contigs were obtained in *S. indica*, *S. tashiroi*, *S. playfairii*, and *S. taiwanense*, respectively, after trimming. Seven *R2R3-MYBs* were identified from the EST contig library of inflorescence bud transcriptomes of *Scutellaria*, including *MYB2/7*, *MYB8*, *MYB11*, *MYB13*, *MYB15*, *MYB16*, and *MYB19*, corresponding with *Arabidopsis* *AtMYB68* (S14), *AtMYB90* (S6), *AtMYB36* (S14), *AtMYB5*, *AtMYB20*, *AtMYB65* (S18), and *AtMYB5*, respectively (Table 3). Among these gene families, both *MYB2/7* and *MYB11*, corresponding to *AtMYB68* (S14), are suggested to be a paralogous relationship; *MYB13* and *MYB19*, corresponding to *AtMYB5*, are also a paralogous relationship*.* These *Scutellaria* *MYB* genes have relatively abundant reads revealed in Fragments Per Kilobase of transcript per Million mapped reads (FPKM) in the transcriptome database, and was suggested to have certain degrees of expression in inflorescence buds of *Scutellaria* species. The closer groupings of these *R2R3-MYBs* expressing in inflorescence buds suggest that the paralogous relationships of these genes could be due to recent duplication in genus *Scutellaria*.

**Table 3:** Grouping of *R2R3-MYBs* by phylogenetic analysis and tBLASTx result by searching to *Arabidopsis thaliana*. The grouping name was based on the *R2R3-MYBs* of *Scutellaria* *baicalensis* [27].

Group Name	Phylogenetic Grouping										tBLASTx to *Arabidopsis*	
	Stas	Spla	Sind	Stai	Sbai	Smon	*Amborella*	*Arabidopsis*	*Solanum*	*Oryza*	Accession Number	*E*-Value
*MYB16*	KP167623	KP167610	KP167603	KP167617	KF008651	ATYL_2013395	–	–	XM_004236340	–	NM_111977	7 × 10^−69^
	(Stas_9284)	(Spla_28842)	(Sind_28842)	(Stai_7654)	(SbMYB16)						(AtMYB65)	
*MYB15*	KP167621	KP167607	KP167598	–	KF008664	ATYL_2028518	XM_006837947	NM_105294	XM_004236642	NM_001070300	NM_105294	1 × 10^−85^
	(Stas_14132)	(Spla_13224)	(Sind_16195)					(AtMYB20)		NM_001054563	(AtMYB20)	
								NM_121666		NM_001063857		
								(AtMYB43)		NM_001068382		
										NM_001069653		
MYB8	KP167618	KP167606	KP167604	KP167613	KF008657	ATYL_2121808	XM_006849579	AF048841	XM_004252468		AF062915	3 × 10^−57^
								(AtMYB82)			(AtMYB90)	
								NM_123397				
								(AtMYB23)		–		
MYB2/7/11							XM_006837947		XM_004245674	NM_001186451		
									XM_004248305			
MYB11	KP167619	KP167609	KP167600	–	KF008660	ATYL_2012934	–	–	–	–	NM_125143	7 × 10^−63^
					(SbMYB11)						(AtMYB36)	
MYB2/7	–	KP167611	KP167605	KP167615	KC990835	ATYL_2108188/2121208	–	–	–	–	AF062901	9 × 10^−71^
					(SbMYB2)						(AtMYB68)	
					KC990836							
					(SbMYB7)							
MYB13/19							XM_006854550	-	XM_004253144			
									XM_004246040			
									XM_004244680			
MYB13	KP167622	KP167608	KP167601	KP167616	KF008662	–	–	–	–	–	NM_112200	3 × 10^−40^
			KP167599		(SbMYB13)						(AtMYB5)	
MYB19	KP167620	KP167612	KP167602	KP167614	KF008667	ATYL_2029067	–	–	–	–	NM_112200	1 × 10^−61^
					(SbMYB19)						(AtMYB5)	

### 2.2. Phylogenetic Analyses

**Figure 1:**
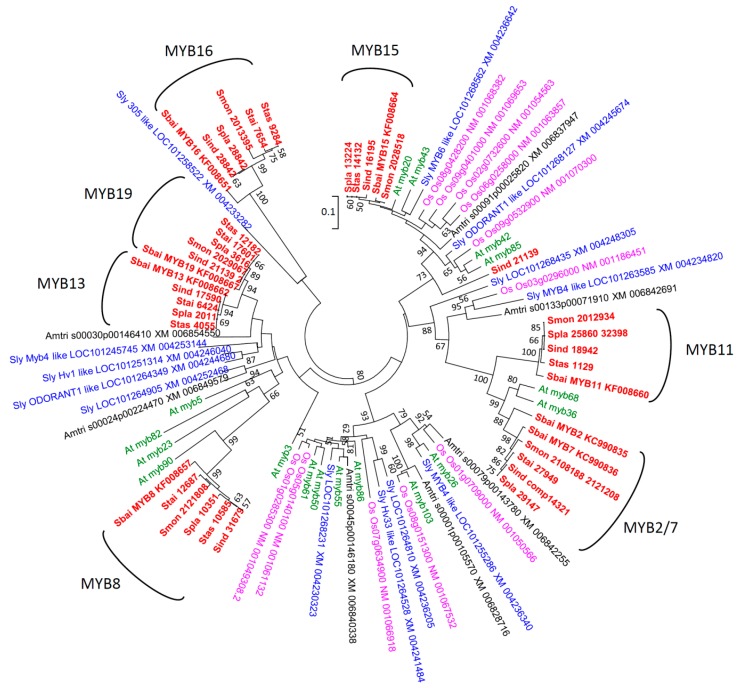
Neighbor-joining (NJ) tree of the *R2R3-MYB* paralogs expressed in the *Scutellaria* inflorescence buds and the corresponding sequences collected from GenBank. The NJ tree is reconstructed from realigned amino-acid sequences chosen from the preliminary NJ analyses (Figure S1). Amtri: *Amborella trichopoda*; At: *Arabidopsis thaliana*; Os: *Oryza sativa*; Sbai: *Scutellaria baicalensis*; Sind: *Scutellaria indica*; Sly: *Solanum lycopersicum*; Smon: *Scutellaria montana*; Spla: *Scutellaria playfairii*; Stai: *Scutellaria taiwanensis*; Stas: *Scutellaria tashiroi*.

Cladograms reconstructed by nucleotide sequences and amino acid sequences revealed inconsistent topologies in basal lineages. Although the relationships of the *R2R3-MYB* paralogs cannot be well resolved, consistent grouping of *Scutellaria* *R2R3-MYBs* with their orthologous genes of *Arabidopsis*, tomato, rice, and *Amborella* were shown in both nucleotide and amino acid trees (Figure S1). The phylogenetic analysis based on the amino acid alignments of conserved region (Figure S2), seven major clades of *Scutellaria* *R2R3-MYBs* are shown in Figure 1 and revealed close grouping between clades *MYB2/7* and *MYB11* and between clades *MYB13* and *MYB19*, congruent with the inference of paralogous relationships made from BLAST searches. However, the phylogenetic grouping patterns among the *Scutellaria* *R2R3-MYBs* and the *AtMYBs* are incongruent with the BLAST results (Table 3), which is probably due to (1) imperfect alignments between diversified taxa and paralogs, and (2) interference by homoplasious codons. The homoplasy is probably due to the long-term evolution of independent lineages having caused a high accumulation of variations between those lineages and, alternatively, may be caused by rapid divergence under positive selection. To investigate the alternative homoplasious cause of selective pressures on genes of recently evolved taxa (*i.e.*, infra-genus species), in which scenario the observed variation was not due to gradual long-term changes, we used the phylogenetic analyses by maximum likelihood (PAML) method to detect signatures of positive selection.

### 2.3. Codon-Specific Positive Selection

**Figure 2:**
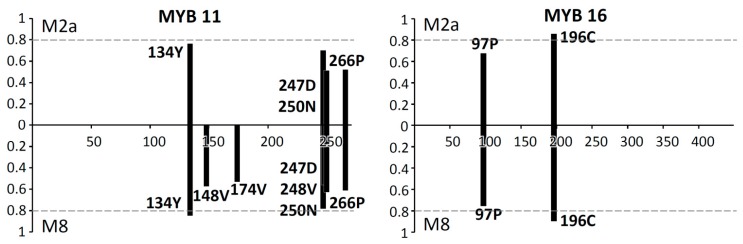
Site-specific profile of the positive selection site models M2a and M8 in *MYB11* and *MYB16*. Horizontal dot line indicates the criterion of posterior probability 0.8.

The site model analyses indicated that the positive selection models M2a and M8 were better fits for *MYB16* by rejecting the null models M1a (*p* = 0.038), M7 (*p* = 0.038), and M8a (*p* = 0.013), and that they were also better fits for *MYB11* by rejecting the null models M1a (*p* = 0.032) and M8a (*p* = 0.048) (Table 4). The site model M7 could not be confidently rejected in favor of M8 in *MYB11* (*p* = 0.082). In *MYB16*, two alignment sites, 97P and 196C, had an estimated ω > 1 in both M2a and M8 models despite relatively low posterior probabilities for 97P (<0.8, Figure 2); in *MYB11*, four and seven codons had an estimated ω > 1 in M2a and M8 models, respectively, but only the 134Y codon in the M8 model had a posterior probability >0.8 (Figure 2). Except for *MYB16* and *MYB11*, no null models (M1a, M7, and M8a) could be rejected by the alternative positive-selection models (M2a and M8) for other *R2R3-MYB* genes by likelihood-ratio tests (LRTs) (*i.e*., *p* > 0.05, Table 4).

**Table 4:** Likelihood ratio tests for the site model analyses in *Scutellaria* *R2R3-MYB* genes. Subgroups of *AtMYB* family defined by Kranz *et al.* (1998) was provided for characterizing the *MYBs* of *Scutellaria* defined by Yuan *et al.* [27].

Model		*AtMYB5-Like*		S6	S14		*AtMYB20*	S18
		*MYB 19*	*MYB 13*	*MYB 8*	*MYB 7*	*MYB 11*	*MYB 15*	*MYB 16*
M1a	lnL	−1869.28	−1419.48	−2050.95	−1685.91	−1763.79	−1803.50	−2587.30
M2a	lnL	−1869.28	−1419.24	−2050.84	−1685.59	−1761.03	−1802.53	−2584.73
	2ΔL	0	0.49	0.22	0.63	5.52	1.94	5.13
	*p*	1	0.391	0.449	0.364	**0.032**	0.19	**0.038**
M7	lnL	−1869.74	−1419.61	−2051.38	−1686.13	−1764.06	−1804.13	−2587.32
M8	lnL	−1869.28	−1419.24	−2050.84	−1685.59	−1762.24	−1802.53	−2584.74
	2ΔL	0.92	0.74	1.07	1.07	3.63	3.2	5.17
	*p*	0.491	0.345	0.293	0.293	0.082	0.101	**0.038**
M8a	lnL	−1869.28	−1419.48	−2050.95	−1685.91	−1763.80	−1803.51	−2587.34
M8	lnL	−1869.28	−1419.24	−2050.84	−1685.59	−1762.24	−1802.53	−2584.74
	2ΔL	0	0.49	0.22	0.63	3.11	1.96	5.19
	*p*	0.5	0.447	0.755	0.365	**0.048**	0.107	**0.013**

*p* values < 0.05 are remarked in bold.

### 2.4. Cluster-Specific Positive Selection and Functional Divergence

For testing whether specific paralogous *R2R3-MYBs* revealed signatures of natural selection, we further performed the clade model test in PAML. The LRTs showed that the nearly neutral model (M1a) was significantly rejected by clade model C in all analyses of different backgrounds (Table 5). The clade *MYB19* was the only foreground clade that has an estimated ω > 1, while the other clades were estimated to have ω < 1 (Table 5). This result indicated that the *Scutellaria* *MYB19* could have been subject to directional selection after gene duplication. In contrast, the *MYB19* paralog, *MYB13*, has extremely low ω (=0.0001), indicating strong purifying selection.

In order to confirm the inference of positive selection on *MYB19* of *Scutellaria*, we used the branch-site model to test whether clade *MYB19* is rapidly evolving in nonsynonymous sites, in contrast to the other *R2R3*-*MYBs*. Significant results of LRT indicated that small fractions of codons (2.72%) of *Scutellaria MYB19* have an estimated ω = 11.1973 (Table 6). The alignment codon site 15K (posterior probability = 0.994) of *MYB19* was the only codon inferred to be positively selected with a posterior probability > 0.95.

**Table 5:** Results of clade model analyses and the likelihood ratio tests.

Background	*Amborella*			*Arabidopsis*			*Oryza*			*Solanum*		
**M1a (Null model)**												
np	101			113			109			121		
lnL	−2402.594			−2695.469			−2735.734			−3170.464		
Site class	Class 0	Class 1		Class 0	Class 1		Class 0	Class 1		Class 0	Class 1	
Proportion	0.938	0.062		0.869	0.131		0.824	0.176		0.99999	0.00001	
ω	0.042	1		0.041	1		0.042	1		0.039	1	
**Clade Model C**												
np	110			122			118			130		
lnL	−2321.832			−2606.658			−2656.505			−3059.730		
Site class	Class 0	Class 1	Class 2	Class 0	Class 1	Class 2	Class 0	Class 1	Class 2	Class 0	Class 1	Class 2
Proportion	0.698	0	0.302	0.710	0	0.290	0.676	0.034	0.290	0.687	0	0.313
*ω_Background_*	0.010	1	0.154	0.013	1	0.183	0.014	1	0.285	0.010	1	0.134
*ω_MYB2/7_*	0.010	1	0.062	0.013	1	0.101	0.014	1	0.040	0.010	1	0.045
*ω_MYB11_*	0.010	1	0.037	0.013	1	0.196	0.014	1	0.097	0.010	1	0.056
*ω_MYB15_*	0.010	1	0.028	0.013	1	0.000	0.014	1	0.017	0.010	1	0.000
*ω_MYB16_*	0.010	1	0.207	0.013	1	0.114	0.014	1	0.085	0.010	1	0.039
*ω_MYB8_*	0.010	1	0.054	0.013	1	0.058	0.014	1	0.029	0.010	1	0.160
*ω_MYB13_*	0.010	1	0.000	0.013	1	0.000	0.014	1	0.000	0.010	1	0.000
*ω_MYB19_*	0.010	1	999	0.013	1	999	0.014	1	999	0.010	1	999
**LRT**												
2ΔL	161.525			177.623			158.457			221.469		
df	9			9			9			9		
*p*	3.58 × 10^−30^			1.59 × 10^−33^			1.55 × 10^−29^			1.03 × 10^−42^		

**Table 6:** Results of branch-site model analysis and the likelihood ratio tests for the foreground branch *MYB19*.

Model	np	lnL	Parameter	Class 0	Class 1	Class 2a	Class 2b
Model A ω = 1 fixed	82	−1486.769	Proportion	0.7630	0.1728	0.0523	0.0119
			Background ω	0.0285	1	0.0285	1
			Foreground ω	0.0285	1	1	1
Model A	83	−1484.552	Proportion	0.7930	0.1798	0.0222	0.0050
			Background ω	0.0292	1	0.0292	1
			Foreground ω	0.0292	1	11.1973	11.1973
LRT ^a^	2ΔL = 4.434, *p* = 0.0176						

^a^ The *p* value is calculated by 50:50 mixture distribution of point mass 0 and χ^2^ with df = 1. Positive sites for foreground lineages Prob (ω > 1): 15K (Prob = 0.994).

**Figure 3:**
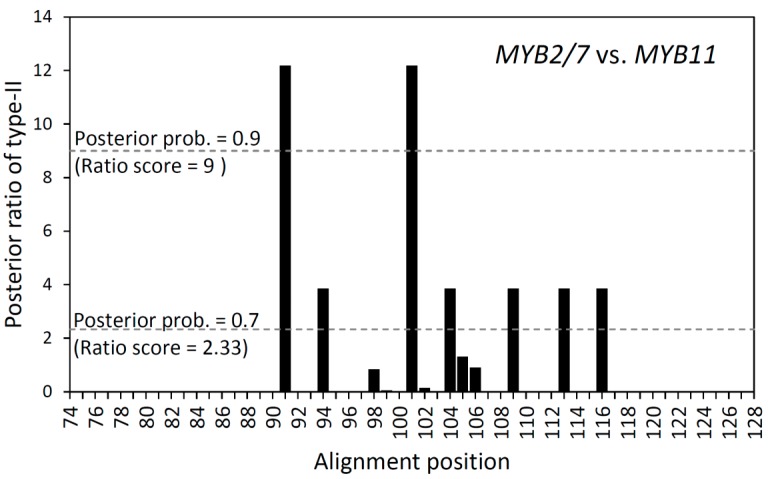
Site-specific profile for type-II functional divergence between *Scutellaria MYB2*/*7* and *MYB11*. Horizontal dot lines indicate the criteria of posterior probability 0.9 and 0.7.

We further performed type-I and type-II functional divergence analyses for the paralogous pair *MYB13* and *MYB19* and another paralogous pair, *MYB2/7* and *MYB11*. Both a Z-score test (*p* < 0.00001) and LRT (*p* = 0.00002) for the estimated θ_I_ showed significant type-I functional divergence between *MYB13* and *MYB19*, but not for the paralogous pair *MYB2/7* and *MYB11* (*p* = 0.099 in Z-score test and 0.956 in LRT, Table 7). The significant type-I divergence between the paralogous pair *MYB13* and *MYB19*, which indicates heterogeneous evolutionary rates after duplication, is consistent with the estimated ω of *MYB13* (ω ≪ 1) and *MYB19* (ω ≫ 1) by clade model C analysis in PAML (Table 6). In contrast, the type-II functional divergence analysis showed significant divergence between *MYB2/7* and *MYB11* by Z-score test (*p* = 0.042) but not for the paralogous pair *MYB13* and *MYB19* (*p* = 0.073) (Table 7). If the indels were not considered, two of 55 aligned amino acid sites (3.64%) of the R2 and R3 domains received a ratio score >9 (*i.e.*, posterior probability > 0.9 or false positive rate < 10%) while seven of 55 alignments (12.73%) received a ratio score >2.33 (*i.e.*, posterior probability > 0.7) specifying cluster-specific radical changes between *MYB2/7* and *MYB11* (Figure 3).

**Table 7:** Summary of type-I and type-II functional divergence.

Type-I Functional Divergence			Type-II Functional Divergence		
Parameter	MYB19 *vs.* MYB13	MYB7/11 *vs.* MYB2	Parameter	MYB19 *vs.* MYB13	MYB7/11 *vs.* MYB2
θ_I_	1.021	−0.413	θ_II_	0.052	0.125
SE θ_I_	0.161	0.293	SE θ_II_	0.036	0.072
*p* of θ_I_ Z-score	**<0.00001**	0.099	*p* of θ_II_ Z-score	0.073	**0.042**
θ_I_ML	0.999	0.006	a_R_/π_R_	1.405	1.869
AlphaML	0.006	0.126	G_R_/G_C_	1	0.864
SE θ_I_	0.234	0.050	F_00,N_	0.927	0.727
LRT θ_I_	18.289	0.003	F_00,R_	0.018	0.036
*p* of LRT θ_I_	**1.898 × 10^−5^**	0.956	F_00,C_	0.018	0.091

a_R_/π_R_: the ratio of radical change under functional divergence *versus* nonfunctional divergence; G_R_ and G_C_, proportion of radical change and conserved change, respectively; F_00,N_, F_00,R_, and F_00,C_, proportion of none change, radical change, and conserved change of amino acids between clusters but no change within clusters, respectively. *p* value < 0.05 is indicated in bold.

## 3. Discussion

### 3.1. Diversification of R2R3-MYBs in Scutellaria Expressed in Inflorescence Buds

*R2R3-MYB* sequences collected from inflorescence bud transcriptomes represent the active (expressed) form of these genes in the flowering programs [35]. Use of tissue-specific transcriptomic library to perform analyses of gene diversification and evolutionary rate heterogeneity highlights the co-expression and functional divergence patterns of these duplicated genes [21,36]. The expression of these duplicated *MYB* genes in the inflorescence buds suggest that these *MYBs* involve in the regulation function of floral pattern or flowering process [34,35]. Most of the *R2R3-MYBs* expressed in the inflorescence have been identified to be involved in the regulation of genes related to anthocyanin biosynthesis; for example, *FtMYB123L*, a homolog of *AtMYB123/TT2* expressed in the flower and inflorescence of *Fagopyrum tataricum*, plays a key role in regulating flavonoid late biosynthetic genes [37]; *LhMYB6* and *LhMYB12*, isolated from the anthocyanin-accumulating tepals of Asiatic hybrid lily (*Lilium* spp.), regulate anthocyanin biosynthesis in flower tepals, tepal spots, and leaves. A recent study indicated that at least two *R2R3-MYBs*, *SbMYB2* and *SbMYB7*, are involved in flavonoid metabolism in *Scutellaria baicalensis* [27]. Most of *SbMYB* genes have fewer paralogs than that found in *Populus* [38]. The *Populus* genome encodes more *R2R3-MYB* family members than either *Arabidopsis* or *Vitis*. Expansion of *R2R3-MYB* family of *Populus* was not only attributable to whole genome duplication but also multiple segmental and tandem duplication events, so that *Populus* genome encodes more *R2R3-MYB* genes than not only other species but also other genes (e.g., *R3-MYBs*) [38,39]. All Taiwanese *Scutellaria* species have same chromosome numbers (2*n* = 26) [8] and no records of genome duplication were reported. In the research performed by Cole *et al.*, both *Scutellaria baicalensis* and *S. racemosa*, which have fewer chromosomes (2*n* = 18), have a smaller genome size (411 and 377 mbp, respectively) than *S. lateriflora* (950 mbp), which has more chromosomes (2*n* = 88) [40]. Diversification and expansion of the *R2R3-MYB* family in *Scutellaria* could be related to the ancestral genome duplication. However, we do not have enough evidence to correlate the genome size or duplicated genomes with the rapid diversification of *R2R3-MYBs* in Taiwanese *Scutellaria* species. Nevertheless, the genome-size effect on the expansion of *R2R3-MYB* family cannot be exclusively ruled out, and it is worth exploring in the future.

### 3.2. Phylogenetic Analyses and Functional Annotation

In the phylogenetic grouping, we found that *SbMYB2* was closely grouped with the clade of *SbMYB7* and other *MYB7* orthologs in *S. indica* (KP167605), *S. playfairii* (KP167611), *S. taiwanensis* (KP167615), and *S. montana* (ATYL 2108188 2121208) with a high bootstrap supporting value (0.97). However, no other sequences were found to be grouped with *SbMYB2*, indicating that either the inflorescence buds of skullcaps do not express orthologs of *MYB2*, or *SbMYB2* is a recent duplication in *S. baicalensis* only. Besides, the *MYB2*/*7* clade was grouped with *SbMYB11* and its orthologs in skullcaps, but was not individually grouped with sequences of *Arabidopsis*, tomato, rice, and *Amborella*, indicating the paralogous relationship of *MYB11* and *MYB2*/*7*. These two paralogous gene families in skullcaps were grouped with S14 of *Arabidopsis thaliana* (Figure 1), which functions in the development of shoot and axillary meristems [28]. *SbMYB2*/*7* and *SbMYB11* were also suggested to function in the regulation of flavonoid biosynthetic pathways and have been confirmed in Tobacco transgenic experiments (Table 2) [27].

In addition, both *MYB13* and *MYB19* of skullcaps, which are named after *SbMYB13* and *SbMYB19*, are grouped with *Odorant1-like* and *Hv1-like* of tomato (Figure 1) and functionally annotated as regulating anthocyanin biosynthesis and pigment accumulation under cold stress (Table 2) [12,29,30]. However, the BLAST search indicates *MYB13* and *MYB19* are similar to *AtMYB5* (Table 3), which is grouped with (Figure 1). The inconsistency between phylogenetic groupings and the BLAST search is probably caused from high degree of homoplasious variations among distinct taxa or few informative sites to sort sequences of same subfamily. Because of the co-expression of paralogous genes in inflorescence buds in skullcaps, we hypothesized that for the prevention of redundancy between duplicated genes, functional divergence would have occurred between the paralogous genes *MYB2*/*7* and *MYB11* and the paralogous pair *MYB13* and *MYB19* in skullcaps.

The BLAST searches and phylogenetic analysis suggested orthology of *MYB8* to AtMYB90 (Figure 1 and Table 3), which functions as the induction of anthocyanin accumulation and expressed in flower buds (Table 2) [28,29]. Besides, tobacco transgenic experiments also confer the function of *SbMYB8* in induction of anthocyanin accumulation [41], and support the view that *SbMYB8* and *AtMYB90* are orthologous.

Although corresponding transgenic line have not been conducted in *MYB15* and *MYB16*, their expression patterns are not consistent with anthocyanin biosynthesis-related genes (e.g., *CHS* and *PAL*) [42], suggesting that *MYB15* and *MYB16* are not involved in regulation of anthocyanins. These two genes may function in the regulation of stress tolerance [32,33] and anther and pollen development [17], respectively (Table 2). The *AtMYB20* promoter, an ortholog of *MYB15*, was found to drive β-glucuronidase (GUS) expression in several tissues including the sepal and style of flowers under the NaCl treatment, and this result was confirmed by RT-PCR experiments [32]. The expression of *MYB15* in skullcaps is probably a response to harsh soil substrates in nature, but more experimental data are needed to confirm the link between *MYB15* and adaptation to stress in skullcaps. *MYB16* is orthologous to *AtMYB65* (S18), which facilitates, but is not essential for, the development of anthers and pollen, with a particular role in the formation of the tapetum, a nutritive layer of pollen grains [17]. The tapetum cells have unique organelle tapetosomes, which secrete flavonoids, alkanes, and oleosins to the surface of the maturing pollen as pollen coats [42], and cause the variation of microtraits on the pollen surface (*i.e.*, exine, Table 1). The variation of pollen surface microtraits might influence the strategy and efficiency of pollination and be related to the reproductive isolation of skullcaps [43].

In general, the *R2R3-MYB* genes expressed in the inflorescence buds have ecological functions related to pollinator attraction and/or response to stresses, thus the genetic variation of these *R2R3-MYB* genes might not be randomly accumulated, but rather be a consequence of natural selection. To verify this hypothesis, we performed further genetic analyses to detect signals of positive selection and test for functional divergence between paralogous genes.

### 3.3. Positive Selection on Scutellaria MYB11 and MYB16

*MYB11* and *MYB16* have relatively high ω (>1) indicating that positive selection could have affected the evolution of these two gene families. *MYB11* functions in the regulation of stem and leaf growth forms and the development of the axillary meristems of inflorescences [14,15], while *MYB16* is functionally relevant to the microRNA regulation of filament development and pollen maturation [17]. According to these functional annotations, we suggested that the high nonsynonymous mutation rates that we found for the *Scutellaria* *MYB11* and *MYB16* might be related to the adaptive divergence of plant growth forms, including the stem/leaf structure and the inflorescence branching pattern, in these studied skullcaps species [14,15,17]. Species of Taiwanese skullcaps, which have been suggested to be rapidly evolving [9], are morphologically varied in petal colors and vegetation forms (Table 1). High interspecific variation in the pollen size and exine, which are major taxonomic characters in *Scutellaria* [44], and which are regulated by *MYB16*, could be related to the pollinator shift between different *Scutellaria* species [43]. The positive sites 134Y in *MYB11* and 196C in *MYB16*, both of which had high posterior probabilities, are located within the DNA-binding domain. Selective constraints usually appeared in functionally important regions, such as the DNA-binding domain [45]. There are three helices in the DNA-binding domain, and the amino acid changes in these helices could cause alteration or loss of protein function [46,47]. Such amino acid replacements in the DNA-binding domain were suggested to be an important mechanism for regulating the diversification of downstream genes and a major driving force for plant diversification [47]. The positive selection on these functionally constrained regions could indicate adaptive changes in protein functions during the evolution of skullcaps. Positive selection on the genes regarding these phenotypic variations suggested the enhancement of the fitness of skullcaps for adaptively relevant environments via increasing the amino acid replacement rates. Floral diversification, which plays key roles in mediating evolutionary transitions and may explain the adaptive radiation of skullcaps, could be driven by shifts between pollinators [48].

### 3.4. Different Types of Functional Divergence between Recent Duplicated Paralogs in Scutellaria R2R3-MYBs

The recently duplicated genes *MYB13*/*MYB19* and *MYB2/7/MYB11*, which are co-expressed in inflorescence buds, were hypothesized to have been subjected to partitioning of ancestral function (sub-F) or functional divergence (neo-F) to prevent functional redundancy after duplication [49,50,51]. We therefore tested the signatures of functional divergence between these two paralogs. Two types of functional divergence, the heterogeneous evolutionary rates between duplicates (type-I) and radical amino acid replacements between duplicates (type-II), were found in *Scutellaria* *MYB13*/*MYB19* and *MYB2/7/MYB11*, respectively (Table 7). We do not know the exact functions of these recently duplicated paralogs due to a lack of physiological and molecular experimental evidence. In other words, we do not understand where (in which tissues) and when (at what stages) these genes are expressed, apart from their known expression in inflorescence buds. However, from the genetic analyses, the significantly different evolutionary rates between *MYB13* and *MYB19* indicated that these simultaneously expressed genes suffered different selective pressures. The accelerated evolutionary rates in *MYB19* could imply the acquisition of novel functions (neo-F) or amplification of a previous neutral minor function (Innovation-Amplification-Divergence (IAD) model), involving positive selection [52,53]. In contrast, its paralogous gene *MYB13*, which was selectively constrained, with extremely low ω, was suggested to preserve ancestral function [52]. Differential selective pressures increase the fitness entropy to move toward novel stationary phases with respect to the surroundings and allow the coexistence of newly derived paralogs.

In contrast to the type-I functional divergence of duplicates *MYB13/MYB19*, evolutionary rate homogeneity was not rejected between duplicates of *MYB2/7* and *MYB11*, but these two duplicates revealed radical amino acid changes (Table 7 and Figure 3). Despite a small ratio of radical change to conserved change (G_R_/G_C_ = 0.864) and only 3.6% fixed radical differences (F_00,R_) being found between these two duplicates, which are the two amino acids at the 91st and 101st alignment sites with posterior probability >0.9 (Figure 3), the relatively small proportion of radical change could potentially have caused the functional divergence. However, despite radical replacements between these two copies, no positive selection signals were detected between *MYB2/7* and *MYB11* (Table 5 and Table 7). Both *MYB2/7* and *MYB11* were subjected to purifying selection with a lower ω than that of the backgrounds (Table 5). The phenomenon of both copies remaining in the genome but having evolved under purifying selection or strong selective constraints could reflect the advantage of dosage effects of functionally redundant duplicates or alternatively reflect their diverged functions [54,55]. If these two duplicated gene pairs were functionally redundant but had an advantageous dosage effect, we could expect that the radical changes would not be found between them. The significant type-II functional divergence found between these duplicates rejects the advantages-of-dosage-effect hypothesis. We suggest that the functional divergence of these two duplicates has already become complex and both paralogs have reached a state of functional constraint. The long-term retention and coexistent expression of duplicates suffering purifying selection is commonly observed in cotton [56] and *Arabidopsis* [57], but most of the duplicated gene pairs exhibit divergent expression between tissues [56]. *MYB2*/*7*/*11* paralogs could function in regulating the expression of several anthocyanin biosynthetic enzymes, including Phenylalanine Ammonia Lyase (PAL), Cinnamate 4-Hydroxylase (C4H), Chalcone Synthase (CHS), Chalcone Isomerase (CHI), and UDP-glucose Flavonoid Glucosyl-Transferase (UFGT) [27]. Functional subdivision is helpful for increasing the efficiency of gene regulation. Although only a small fraction of functionally divergent codons was found (Figure 3), these radical changes could have adaptive relevance for the floral diversity. Because we pooled several samples of the same species to obtain the consensus gene sequences instead of individually sequencing transcriptomes, the relative expression level of paralogs cannot be obtained by counting sequence reads (*i.e.*, fragments per kb of transcript per million mapped reads), and thus the potential existence of a gene dosage or genetic buffering effect could not be confirmed by this study. However, this research provides statistical evidence to illustrate the radical functional divergence of the *MYB2*/*7* and *MYB11* paralogs. Further manipulative experiments are required to elucidate the evolutionary and functional processes of the retention and co-expression of these duplicated genes.

## 4. Experimental Section

### 4.1. Data Collection, de Novo Transcriptome Assembly, and Annotation

Transcriptomic libraries of the inflorescence buds of *S. taiwanensis*, *S. indica*, *S. playfairii*, and *S. tashiroi* were constructed by transcriptome *de novo* sequencing by Illumina Solexa technology. For enriching the sequence data, the published shoot transcriptome data of *S. montana* were down-loaded from the 1KP database (Accession ID: ATYL). Removing artifacts from RNA-Seq reads before assembly can improve the accuracy and computational efficiency of assembly [58]. Therefore, raw RNA-Seq reads were controlled by quality score. Bad quality reads (score < 20) were trimmed using our PERL scripts. Then, reads that have length ≥25 bps on both sides of paired-end format were kept for further analyses. Filtered reads were assembled *de novo* using Trinity (Trinityrnaseq_r2013-02-25) [59]. Trinity was used with the defaulting parameters including a fixed *k-*mer at 25 bp.

Eleven *MYBs* identified as the *R2R3-MYBs* from *S. baicalensis* were used as references for local BLAST alignment to our expressed sequence tag (EST) contig libraries of inflorescence-bud transcriptomes. Sequences of the first three highest *e*-values with >50% coverage were sampled. Putative *R2R3-MYBs* of skullcaps were reconfirmed by a bidirectional best hit (BBH) approach and BLAST alignment to the sequences obtained from the NCBI GenBank database. The confirmed *R2R3-MYBs* of *Scutellaria* were further aligned using the MUSCLE multiple sequence alignment software tool [60,61]. *Arabidopsis*, tomato, rice, and *Amborella* *R2R3-MYBs* were used for neighbor-joining (NJ) comparison with these putative *R2R3-MYBs* of *Scutellaria* to confirm their orthology. The Jones–Taylor–Thornton substitution model with a 95% coverage cutoff, partial deletion, and 1000 times bootstrap replicates was used for NJ analysis. Orthologous sequences of *Scutellaria R2R3-MYB* genes that expressed in inflorescence buds and the *Arabidopsis*, tomato, rice, and *Amborella* *R2R3-MYB* orthologs were further selected to reconstruct fine-scale evolutionary trees by the NJ method. Sequences used in this study were deposited in the NCBI database (Accession number: KP167598–KP167623).

### 4.2. Detecting Positive Selection

Heterogeneous nonsynonymous and synonymous substitution rates (d_N_ and d_S_) were estimated to detect selective pressures using the codeml program distributed with the PAML software package [62]. An excess of d_N_ indicates rapid amino acid replacements and implies positive selection on the gene, and in this case the d_N_/d_S_ ratio, denoted as ω, would be >1; in contrast, an excess of d_S_ indicates the elimination of disadvantage replacements, suggesting purifying selection or selective constraints (ω < 1); if d_N_ = d_S_ (or ω = 1); this means that silent and missense mutations appeared randomly, *i.e.*, the gene evolved neutrally. Likelihood ratio tests (LRTs) were used to evaluate the better fitting model from the comparison pairs of M1a (nearly neutral) *vs.* M2a (positive selection), M7 (the β model) *vs.* M8 (beta plus positive selection model), M8a (beta and ω = 1) *vs.* M8 in site model test, and M1a *vs.* model C in clade model. For the clade model C, each *Scutellaria R2R3-MYB* subfamily was specified as an independent foreground clade and compared with background, comprising *Arabidopsis*, tomato, rice, and *Amborella*, to test which specific subfamily has the most rapid replacement rate for diversifying the regulation of anthocyanin biosynthesis in inflorescence buds of *Scutellaria* species. The specific clade that has higher ω (*i.e.*, ω > 1) was further set as the foreground branch in branch-site model analysis to identify codons that are putatively under positive selection. When performing the branch-site model analysis, we used the *Scutellaria* sequences only to prevent high saturation in nucleotide replacement that may bias the selection analyses. Branch-site model A was used for comparison with the modified null model A with a corresponding fixed value of ω_2_ = 1. For obtaining convergence results, we performed the site-model, clade-model, and branch-site-model analyses multiple times using different initial ω and the number of categories of NSsite model (ncatG). Because all runs showed convergent inferences of positive selection signals, the results with the simplest setting was adopted in this study.

### 4.3. Functional Divergence Analyses

Function divergence between two paralogous pairs, *MYB2*/*7 vs.* *MYB11* and *MYB13 vs.* *MYB19*, was inferred by type-I (Gu99) and type-II divergence analyses using the software program DIVERGE version 3 [63] with 500 bootstrap replications. Type-I functional divergence suggests heterogeneous evolutionary rates between duplicated genes, while type-II functional divergence suggests radical changes to biochemical properties (charge positive/negative, hydrophilic/hydrophobic) between duplicates. Site-specific estimation of the posterior probability of radical changes was performed to assess the probable regions and shifts of biochemical properties between paralogous groups. The posterior ratio given by the equation *R*(*k*) = *Q*(*k*)/[1 − *Q*(*k*)] was used to calculate the posterior probability of sites with type-II divergent functions, where Q is the specific score for site *k* related to type-II functional divergence [64].

## 5. Conclusions

In this research, signals of positive selection were detected in two *MYB* genes that were co-expressed in the inflorescence buds of *Scutellaria* species, in contrast to the previous reports of relaxed selective constraints on anthocyanin-regulating transcription factors [45,65,66]. This finding showed drastic differences in evolutionary rates and radical amino acid changes between *R2R3-MYB* duplicates in rapidly evolving *Scutellaria* species. Regulatory genes exhibit complicated evolutionary mechanisms responsible for morphological diversity, which are relevant to adaptation and speciation [67,68]. This study not only presents evidence for rapid and diversified evolution in *Scutellaria* *R2R3-MYBs* but also indicates the significance of the duplication and sub-F/neo-F of regulatory genes for the diversification of adaptive traits.

## Supplementary Materials

Supplementary materials can be found at: http://www.mdpi.com/1422-0067/16/03/5900/s1.
