# Supplementary material for: Positive Selection and Functional Divergence of R2R3-MYB Paralogous Genes Expressed in Inflorescence Buds of Scutellaria Species (Labiatae)
Source: Int J Mol Sci. 2015 Mar 13;16(3):5900–21. doi: 10.3390/ijms16035900 (PMC4394512; doi:10.3390/ijms16035900)
Supplement: Supplementary file 1 [file ijms-16-05900-s001.pdf]

Supplementary Information

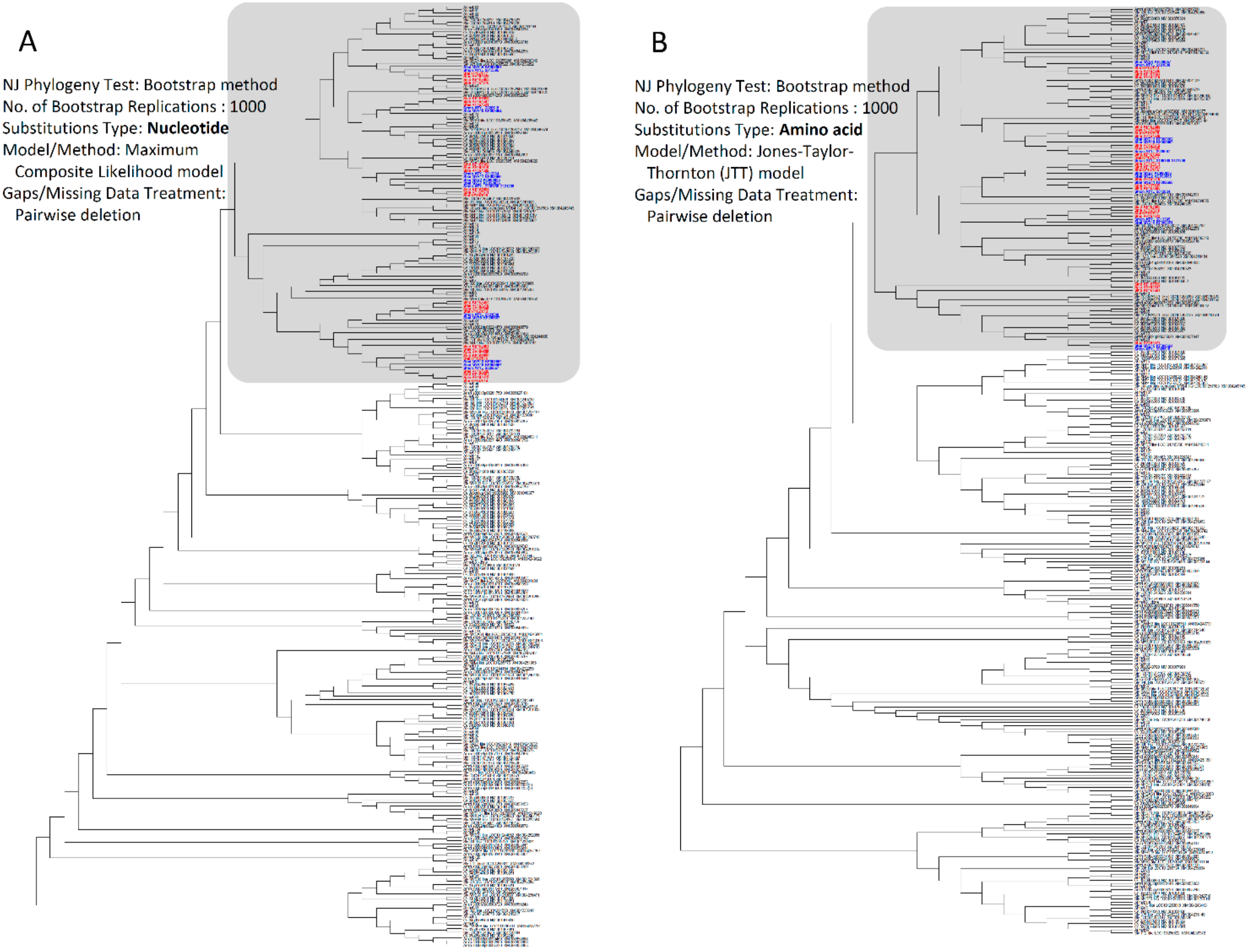

Figure S1. *Cont.*

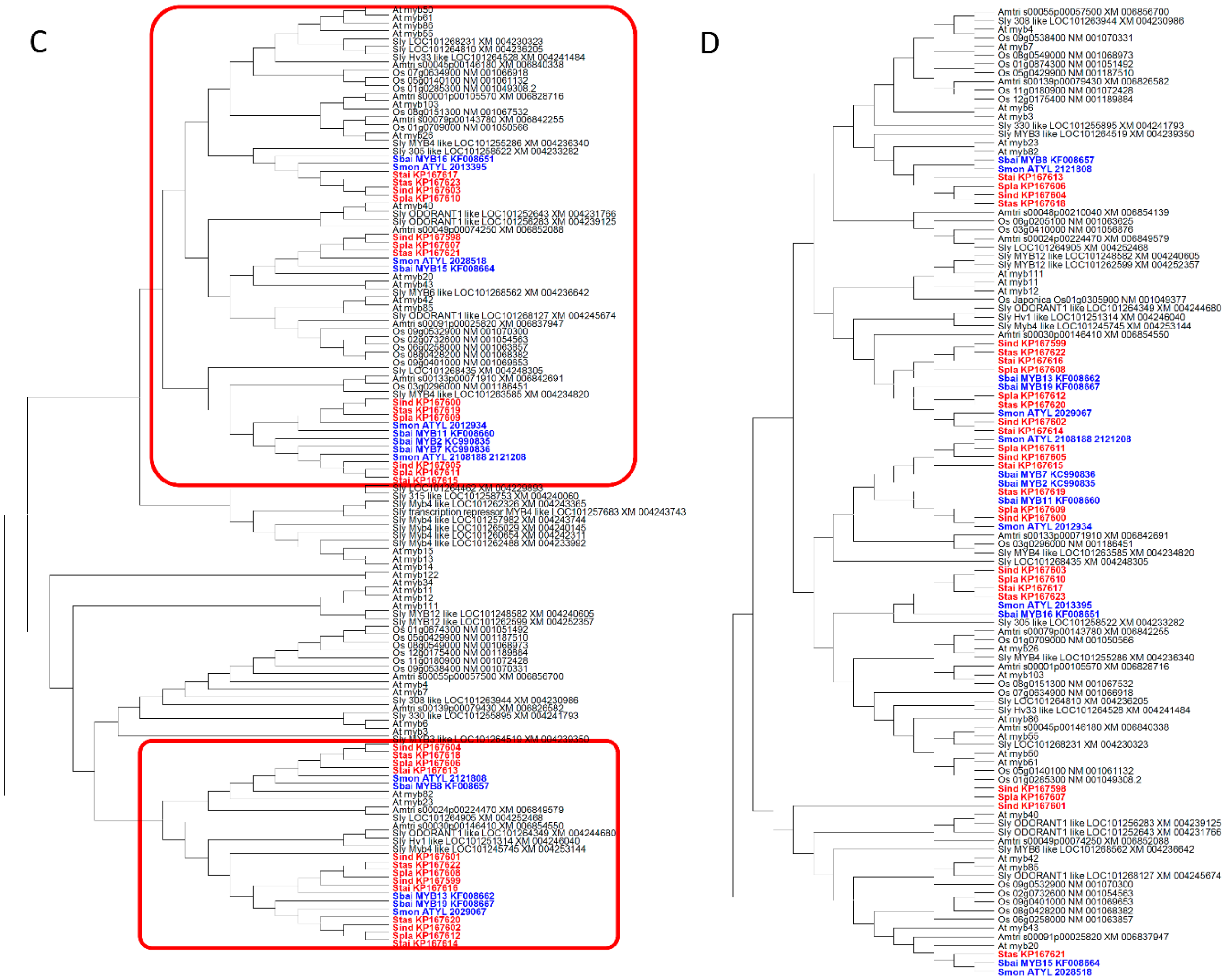

Figure S1. Cladogram of the *Scutellaria* R2R3-MYBs extracted from the inflorescence-bud transcriptomic library of *Scutellaria* and the published R2R3-MYBs from GenBank. (**A**) Neighbor-joining tree reconstructed based on the nucleotide sequences; (**B**) Neighbor-joining tree reconstructed based on the amino acid sequences. The gray boxes are the clade including the *Scutellaria* R2R3-MYBs and the corresponding sequences of *Amborella trichopoda* (Amtri), *Arabidopsis thaliana* (At), *Oryza sativa* (Os), and *Solanum lycopersicum* (Sly); (**C**) and (**D**) are enlargements of the gray areas of Figure S1A,B, respectively. Based on Figure S1C, the *Scutellaria* R2R3-MYBs and the sequences that were grouped with *Scutellaria* R2R3-MYBs were selected (the red frames) and realigned to re-perform the NJ analysis shown in Figure 1. Sbai: *Scutellaria baicalensis*; Sind: *Scutellaria indica*; Smon: *Scutellaria montana*; Spla: *Scutellaria playfairii*; Stai: *Scutellaria taiwanensis*; Stas: *Scutellaria tashiroi*.

```
                                  ....|....| ....|....| ....|....| ....|....| ....|....| ....|....| ....|....| ....|....| ....|....| ....|....| ....|..
                                      5         15         25         35         45         55         65         75         85         95        105
Amtri_s00001p00105570_XM_006828716 MGHHSCCNQQ KVKRGLWSPE EDEKLVRYIT SNGYG-CWSE VPEKAGLQRC GKSCRLRWIN YLRPDIRRGR FTSEEEKIII NLHAVVGNRW AHIASHLPGR TDNEIKN
Amtri_s00024p00224470_XM_006849579 MGRCPCCSKE GLNKGAWTAQ EDKILTDYIK THGEG-KWRT LSQSTGLKRC GKSCRLRWLN YLRPDIKRGN FSDQEEDLII RLHKLLGNRW SLIAGRLPGR TDNEIKN
Amtri_s00030p00146410_XM_006854550 MGRAPCCSKV GLLRGPWIAR EDTLLKRYIE THGEG-RWRS LPKKAGLLRC GKSCRLRWMN YLRPNIKRGN IKQDEEELIV RLHALLGNRW SLIAGRLPGR TDNEIKN
Amtri_s00045p00146180_XM_006840338 MGRHSCCYKQ KLRKGLWSPE EDEKLLRHIT KYGHG-CWSS VPKQAGLQRC GKSCRLRWIN YLRPDLKRGT FSQQEENMII ELHAVLGNRW SQIAAQLPGR TDNEIKN
Amtri_s00079p00143780_XM_006842255 MGHHSCCNKQ KVRRGLWSPE EDEKLINHIA TYGHG-CWSS VPRQAGLQRC GKSCRLRWIN YLRPDLKRGN FSPEEESLII DLHRMLGNRW AQIAKHLPGR TDNEVKN
Amtri_s00091p00025820_XM_006837947 MGRQPCCDKV GLKKGPWTAD EDKKLINFIL TNGQC-CWRA VPKLAGLLRC GKSCRLRWTN YLRPDLKRGL LSESEEQMVI DIHAYLGNRW SKIAAHLPGR TDNEIKN
Amtri_s00133p00071910_XM_006842691 MGRPPCCDKS SVRKGPWTPE EDAKILAYIS GHGTG-NWTA VPKKAGLKRC GKSCRLRWTN YLRPDLKHDR FTPQEEDLIV SLHAAIGSRW SLIAAHLPGR TDNDIKN
Os_Os01g0285300_NM_001049308.2     MGRHSCCYKQ KLRKGLWSPE EDEKLMNHIT KHGHG-CWST VPKLAGLQRC GKSCRLRWIN YLRPDLKRGA FSQEEEDLIV ELHAVLGNRW SQIATRLPGR TDNEIKN
Os_Os01g0709000_NM_001050566       MGHHSCCNKQ KVRRGLWSPE EDEKLVKYIS THGHG-CWSS VPRLAGLQRC GKSCRLRWIN YLRPDLKRGS FSQQEESLII ELHRVLGNRW AQIAKHLPGR TDNEVKN
Os_Os02g0732600_NM_001054563       MGRQPCCEKV GLKKGPWTAD EDQKLVTFLL SNGHC-CWRL VPKLAGLLRC GKSCRLRWTN YLRPDLKRGL LSEEEEKLVI DLHEQLGNRW SKIAARLPGR TDNEIKN
Os_Os03g0296000_NM_001186451       MGRPPCCDKA NVKKGPWTAE EDAKLLAYTS THGTG-NWTS VPQRAGLKRC GKSCRLRYTN YLRPNLKHEN FTQEEEELIV TLHAMLGSRW SLIANQLPGR TDNDVKN
Os_Os05g0140100_NM_001061132       MGRHSCCYKQ KLRKGLWSPE EDEKLMNHIT KHGHG-CWSS VPKLAGLQRC GKSCRLRWIN YLRPDLKRGA FSQEEEDLII ELHAVLGNRW SQIAAQLPGR TDNEIKN
Os_Os06g0258000_NM_001063857       MGRQPCCDKV GLKKGPWTAE EDQKLVAFLL THGHC-CWRV VPKLAGLLRC GKSCRLRWTN YLRPDLKRGL LSDDEERLVI DLHAQLGNRW SKIAARLPGR TDNEIKN
Os_Os07g0634900_NM_001066918       MAKRAGGARK KLRRGLWSPE EDEKLMNHIA KYGHG-CWSS VPKLAGLERC GKSCRLRWIN YLRPDLKRGA FSQEEEDLII HLHSMLGNKW SQIAAQLPGR TDNEVKN
Os_Os08g0151300_NM_001067532       MGHHSCCNQQ KVKRGLWSPE EDEKLIRYIT THGYG-CWSE VPEKAGLQRC GKSCRLRWIN YLRPDIRRGR FTAEEEKLII SLHAIVGNRW AHIASHLPGR TDNEIKN
Os_Os08g0428200_NM_001068382       MGRQPCCDKV GLKKGPWTAE EDQKLITFLL TNGQC-CWRA VPKLAGLLRC GKSCRLRWTN YLRPDLKRGL LSDAEEKIVV DLHAQLGNRW SKIASHLPGR TDNEIKN
Os_Os09g0401000_NM_001069653       MGRQPCCDKV GLKKGPWTAE EDQKLVSFLL GNGQC-CWRA VPKLAGLLRC GKSCRLRWTN YLRPDLKRGL LSETEEKTVI DLHEQLGNRW SKIASHLPGR TDNEIKN
Os_Os09g0532900_NM_001070300       MGRQPCCDKL GVKRGPWTAE EDKKLMSFIL TNGHC-CWRA VPKLAGLLRC GKSCRLRWTN YLRPDLKRGL LTDAEEQLVI DLHAKLGNRW SKIAAKLPGR TDNEIKN
At_myb103                          MGHHSCCNQQ KVKRGLWSPE EDEKLIRYIT THGYG-CWSE VPEKAGLQRC GKSCRLRWIN YLRPDIRRGR FSPEEEKLII SLHGVVGNRW AHIASHLPGR TDNEIKN
At_myb20                           MGRQPCCDKV GLKKGPWTAE EDRKLINFIL TNGQC-CWRA VPKLSGLLRC GKSCRLRWTN YLRPDLKRGL LSDYEEKMVI DLHSQLGNRW SKIASHLPGR TDNEIKN
At_myb23                           MRMTRDGKEH EYKKGLWTVE EDKILMDYVR THGQG-HWNR IAKKTGLKRC GKSCRLRWMN YLSPNVNRGN FTDQEEDLII RLHKLLGNRW SLIAKRVPGR TDNQVKN
At_myb26                           MGHHSCCNKQ KVKRGLWSPE EDEKLINYIN SYGHG-CWSS VPKHAGLQRC GKSCRLRWIN YLRPDLKRGS FSPQEAALII ELHSILGNRW AQIAKHLPGR TDNEVKN
At_myb36                           MGRAPCCDKA NVKKGPWSPE EDVKLKDYID KYGTGGNWIA LPQKIGLKRC GKSCRLRWLN YLRPNIKHGG FSEEEDRIIL SLYISIGSRW SIIAAQLPGR TDNDIKN
At_myb42                           MGRQPCCDKL MVKKGPWTAE EDKKLINFIL TNGHC-CWRA LPKLAGLRRC GKSCRLRWTN YLRPDLKRGL LSDAEEQLVI DLHALLGNRW SKIAARLPGR TDNEIKN
At_myb43                           MGRQPCCDKV GLKKGPWTIE EDKKLINFIL TNGHC-CWRA LPKLSGLLRC GKSCRLRWIN YLRPDLKRGL LSEYEEQKVI NLHAQLGNRW SKIASHLPGR TDNEIKN
At_myb5                            KKTTPCCTKM GMKRGPWTVE EDEILVSFIK KEGEG-RWRS LPKRAGLLRC GKSCRLRWMN YLRPSVKRGG ITSDEEDLIL RLHRLLGNRW SLIAGRIPGR TDNEIKN
At_myb50                           MKRHSCCYKQ KLRKGLWSPE EDEKLLNYIT KHGHG-CWSS VPKLAGLERC GKSCRLRWIN YLRPDLKRGA FSSEEQNLIV ELHAVLGNRW SQIAARLPGR TDNEIKN
At_myb55                           MGRHSCCYKQ KLRKGLWSPE EDEKLLRYIT KYGHG-CWSS VPKQAGLQRC GKSCRLRWIN YLRPDLKRGA FSQDEENLII ELHAVLGNRW SQIAAQLPGR TDNEIKN
At_myb61                           MGRHSCCYKQ KLRKGLWSPE EDEKLLTHIT NHGHG-CWSS VPKLAGLQRC GKSCRLRWIN YLRPDLKRGA FSPEEENLIV ELHAVLGNRW SQIASRLPGR TDNEIKN
At_myb68                           MGRAPCCDKA NVKKGPWSPE EDAKLKDYIE NSGTGGNWIA LPQKIGLRRC GKSCRLRWLN YLRPNIKHGG FSEEEDNIIC NLYVTIGSRW SIIAAQLPGR TDNDIKN
At_myb82                           MECKREEGKS YVKRGLWKPE EDMILKSYVE THGEG-NWAD ISRRSGLKRG GKSCRLRWKN YLRPNIKRGS MSPQEQDLII RMHKLLGNRW SLIAGRLPGR TDNEVKN
At_myb85                           MGRQPCCDKL GVKKGPWTVE EDKKLINFIL TNGHC-CWRA LPKLAGLRRC GKSCRLRWTN YLRPDLKRGL LSHDEEQLVI DLHANLGNKW SKIASRLPGR TDNEIKN
At_myb86                           MGRHSCCFKQ KLRKGLWSPE EDEKLLNYIT RHGHG-CWSS VPKLAGLQRC GKSCRLRWIN YLRPDLKRGA FSQDEESLII ELHAALGNRW SQIATRLPGR TDNEIKN
Sly_LOC101258522_XM_004233282      -------QDV EVRKGPWTME EDLILINYIA NHGEG-VWNS LAKSAGLKRT GKSCRLRWLN YLRPDVRRGN ITPEEQLLIM ELHAKWGNKW SKIAKHLPGR TDNEIKN
Sly_LOC101251314_XM_004246040      MGRAPCCAKE GLRKGPWSTK EDLLLTNYIN QYGEG-QWRS LPKNAGLLRC GKSCRLRWVN YLKPGIKRGN FSEDEEDLIV RLHSLLGNRW SLIAGRLPGR TDNEIKN
Sly_LOC101264528_XM_004241484      MGRHSVFVKE KTRKGLWSPE EDEKLYNYIT RFGVG-CWSS VPKLAGLQRC GKSCRLRWIN YLRPDLKRGM FSQEEEDMII TLHKVLGNRW AQIAAKLPGR TDNEIKN
Sly_LOC101264810_XM_004236205      MGRHSCSVKQ KLRKGLWSPE EDEKLSNYIT NFGIG-SWSS VPKLAGLQRC GKSCRLRWIN YLRPDLKRGM FSQDEEDKII SLHQVLGNRW AQIAAQLPGR TDNEIKN
Sly_LOC101264905_XM_004252468      MGRKPCCSKE GLNKGAWTPL EDKILMDYIK VHGEG-KWRN LPKGAGLKRC GKSCRLRWLN YLRPDIKRGN ISPDEEDLII RLHKLLGNRW SLIAGRLPGR TDNEIKN
Sly_LOC101268231_XM_004230323      MGRHSCCYKQ KLRKGLWSPE EDEKLIKHIT KFGHG-CWSS VPKLAGLQRC GKSCRLRWIN YLRPDLKRGT FSQDEENLII ELHAVLGNKW SQIAARLPGR TDNEIKN
Sly_LOC101268435_XM_004248305      MGRIPCCEKD NVKRGQWTPE EDHKLSSYIA QHGTR-NWRL IPKHAGLQRC GKSCRLRWTN YLRPDLKHGQ FSEAEEQTIV TLHSVLGNRW SVIAAQLPGR TDNDVKN
Sly_LOC101245745_XM_004253144      MGRTPCCSKV GMKKGPWSIE EDMLLTSYIH LHGEG-NWRS LPMNAGLLRC SKSCRLRWVN YLRPGIKRGN FSPEEDDLII PLYSLLGGRW SLIAGRLSGR TDNEIKN
Sly_LOC101255286_XM_004236340      MGHHTCCNKQ KVKRGLWSPE EDEKLINYIT TYGHA-CWSS VPKLAGLQRC GKSCRLRWIN YLRPDLKRGS FSPQEAALII ELHGILGNRW AQIAKHLPGR TDNEVKN
Sly_LOC101263585_XM_004234820      MGRPPCCDKA NVKRGPWTAE EDAKILAYVA THGIG-NWTL VPQKAGLNRC GKSCRLRWTN YLRPDLKHDN FTPQEEDCIL ELHKTIGSRW SLIAKQLPGR TDNDVKN
Sly_LOC101268562_XM_004236642      MGRQPCCDKN EVKKGPWSSE EDKKLTNFIL NNGQC-CWRS LPKLAGLLRC GKSCRLRWTN YLRPDLKRGL LSEYEEKMVI DLHAQLGNRW SKIASHLPGR TDNEIKN
Sly_LOC101264349_XM_004244680      MGRAPCCAKE GLRKGPWSTN EDLLLTNYIK ENGEG-QWRN LPNKAGLLRC GKSCRLRWMN YLRPGIKRGN FSQDEEDLII RLHSLLGNRW SLIAGRLPGR TDNEIKN
Sly_LOC101268127_XM_004245674      MGRQPCCDKL GVKKGPWTAE EDKKLINFIL TNGQC-CWRA VPKLAGLRRC GKSCRLRWTN YLRPDLKRGL LSEAEEKLVI DLHARLGNRW SKIAARLPGR TDNEIKN
Smon_2012934                       MGRAPCCDKT KVKRGPWSPE EDNILKNYVQ QHGTSGSWIA LPLKAGLKRC GKSCRLRWLN YLRPNIKHGG FTEEEDNIIL TLYHNIGTRW SVIASQLPGR TDNDIKN
Smon_2013395                       ANSGENVGEN TLKKGPWTSS EDDILIEYVT KHGEG-NWNA VRKHTGLARC GKSCRLRWSN HLRPDLNKEA FSPEEKHRII ELHAKMGNKW ARMAAELPGR TDNEIKN
Smon_2028518                       MGRQPCCDKV GLKKGPWTAE EDKKLINFIL TNGQC-CWRA VPKLAGLLRC GKSCRLRWTN YLRPDVKRGL LSDYEEKMVI DLHAQLGNRW SKIASHLPGR TDNEIKN
Smon_2029067                       MGRSPCCSKV GLKRGPWSTK EDTLLANYIH QHGEG-QWRS LPKQAGLLRC GKSCRLRWMN YLRPGIKRGN ISEDEEDLIV RLHGLLGNRW SLIAGRLPGR TDNEIKN
Smon_2108188_2121208               MGRAPCCDKA NVKKGPWSPE EDAQLKQYME KHGTGGNWIA LPHKAGLRRC GKSCRLRWLN YLRPNIKHGE FTDEEDRIIC SLFTRIGSRW SIIAGQLPGR TDNDIKN
Smon_2121808                       MEKKGE---- -VKRGSWTKE EDTLLRICID KFGEG-KWHK VPIRAGLNRC RKSCRLRWMN YLRPNIKRGY FTKDEVDLIQ RLHKLLGNRW SLIAGRLPGR TANDVKN
Sbai_MYB11_KF008660                MGRAPCCDKT KVKRGPWSPE EDNILKNYVQ QHGTSGSWIA LPLKAGLKRC GKSCRLRWLN YLRPNIKHGG FTEEEDHTIL ALYHNIGTRW SVIASQLPGR TDNDIKN
Sbai_MYB13_KF008662                MGRSPCCSKV GLKRGPWSTK EDTLLTNFIH QNGEG-QWRS LPKKAGLLRC GKSCRLRWMN YLRPGIVRGN ITEDEEDLIV RLHALLGNRW SLIAGRLPGR TDNEIKN
Sbai_MYB15_KF008664                MGRQPCCDKV GLKKGPWTAE EDKKLINFIL TNGQC-CWRA VPKLAGLLRC GKSCRLRWTN YLRPDVKRGL LSDYEEKIVI DLHAQLGNRW SKIASHLPGR TDNEIKN
Sbai_MYB16_KF008651                ANSGGNIGES NLKKGPWTLS EDEILVEYVT KHGEG-NWNA VQKHTGLARC GKSCRLRWAN HLRPDLKKGA FSPEEEPLII ELHARMGNKW ARMAAELPGR TDNEIKN
Sbai_MYB19_KF008667                MGRSPCCSKV GLKRGPWSTK EDTLLVNYIH QHGEG-QWRS LPKQAGLLRC GKSCRLRWMN YLRPGIKRGN ISEDEEDLIV RLHGLLGNRW SLIAGGLPGR TDNEIKN
Sbai_MYB2_KC990835                 MGRAPCCDKG NVKKGPWSPE EDAKLKEYIE KNGTGGNWIA LPHKVGLRRC GKSCRLRWLN YLRPNIKHGE FSDDEDRIIC TLFATIGSRW SIIAGSLPGR TDNDIKN
Sbai_MYB7_KC990836                 MGRAPCCDKA NVKKGPWSPE EDAQLKDYME KHGTGGNWIA LPHKAGLKRC GKSCRLRWLN YLRPNIKHGE FTDEEDRIIC SLFARIGSRW SIIAGQLPGR TDNDIKN
Sbai_MYB8_KF008657                 MEKKGV---- -VKRGAWTKE EDTLLRICIE KFGEG-KWHK VPIRAGLNRC RKSCRLRWMN YLRPNIKRGY FTKDEVDLIQ RLHKLLGNRW SLIAGRLPGR TANDVKN
Sind_16195                         MGRQPCCDKV GLKKGPWTAE EDKKLINFIL TNGQC-CWRA VPKLAGLLRC GKSCRLRWTN YLRPDVKRGL LSEYEEKMVI DLHAQLGNRW SKIASNLPGR TDNEIKN
Sind_17590                         MGRSPCCSKV GLKRGPWSTK EDTLLTNFIH QNGEG-QWRS LPKKAGLLRC GKSCRLRWMN YLRPGIVRGN ITEDEEDLIV RLHGLLGNRW SLIAGRLPGR TDNEIKN
Sind_18942                         MGRAPCCDKT KVKRGPWSPE EDNILKNYVQ QHGTSGSWIA LPLKAGLKRC GKSCRLRWLN YLRPNIKHGG FTEEEDNIIL TLYHNIGTRW SVIASQLPGR TDNDIKN
Sind_21139                         MGRKPCCDKV GLKKGSWTAD EDKKLINFML TNGQC-CWRA VPKLAGLMRC GKSCRLRWMN YLRPGIKRGN ISEDEEDLIV RLHGLLGNRW SLIAGRLPGR TDNEIKN
Sind_21139_2                       MGRSPCCSKV GLKRGPWSTK EDTLLANYIH QHGEG-QWRS LPKQAGLLRC GKSCRLRWMN YLRPGIKRGN ISEDEEDLIV RLHGLLGNRW SLIAGRLPGR TDNEIKN
Sind_28842                         ANSGENVGEN TLKKGPWTSS EDDILIDYIT KHGEG-NWNA VRKHTGLARC GKSCRLRWAN HLRPDLKKEA FSPEEEYRII ELHAKMGNKW ARMAAELPGR TDNEIKN
Sind_31679                         MEKRAE---- -VKRGAWTKE EDTLLRICID KFGEG-KWHK VPIRAGLNRC RKSCRLRWMN YLRPNIKRGY FTKDEVDLIQ RLHKLLGNRW SLIAGRLPGR TANDVKN
Sind_comp14321                     MGRAPCCDKA NVKKGPWSPE EDAQLKEYME KHGTGGNWIA LPHKAGLRRC GKSCRLRWLN YLRPNIKHGE FTDEEDRTIC SLFARIGSRW SIIAGQLPGR TDNDIKN
Spla_10351                         MENRAE---- -VKRGAWTKE EDTLLRICID KFGEG-KWHK VPIRAGLNRC RKSCRLRWMN YLRPNIKRGY FTKDEVDLIQ RLHKLLGNRW SLIAGRLPGR TANDVKN
Spla_13224                         MGRQPCCDKV GLKKGPWTAE EDKKLINFIL TNGQC-CWRA VPKLAGLLRC GKSCRLRWTN YLRPDVKRGL LSEYEEKMVI DLHAQLGNRW SKIASHLPGR TDNEIKN
Spla_2011                          MGRSPCCSKV GLKRGPWSTK EDTLLTNFIH QNGEG-QWRS LPKKAGLLRC GKSCRLRWMN YLRPGIVRGN ITEDEEDLIV RLHGLLGNRW SLIAGRLPGR TDNEIKN
Spla_25860_32398                   MGRAPCCDKT KVKRGPWSPE EDNILKNYVQ QHGTSGSWIA LPLKAGLKRC GKSCRLRWLN YLRPNIKHGG FTEEEDNIIL TLYHNIGTRW SVIASQLPGR TDNDIKN
Spla_28842                         ANSGENVGEN TLKKGPWTSS EDDILIDYIT KHGEG-NWNA VRKHTGLARC GKSCRLRWAN HLRPDLKKEA FSPEEEYRII ELHAKMGNKW ARMAAELPGR TDNEIKN
Spla_29147                         MGRAPCCDKA NVKKGPWSPE EDAQLKEYME KHGTGGNWIA LPHKAGLRRC GKSCRLRWLN YLRPNIKHGE FTDEEDRTIC SLFARIGSRW SIIAGQLPGR TDNDIKN
Spla_3616                          MGRSPCCSKV GLKRGPWSTK EDTLLANYIH QHGEG-QWRS LPKQAGLLRC GKSCRLRWMN YLRPGIKRGN ISEDEEDLIV RLHGLLGNRW SLIAGRLPGR TDNEIKN
Stai_12687                         MEKKAE---- -VKRGAWTKE EDTLLRICID KFGEG-KWHK VPIRAGLNRC RKSCRLRWMN YLRPNIKRGY FTKDEVDLIQ RLHKLLGNRW SLIAGRLPGR TANDVKN
Stai_17601                         MGRSPCCSKV GLKRGPWSTK EDTLLANYIH QHGEG-QWRS LPKQAGLLRC GKSCRLRWMN YLRPGIKRGN ISEDEEDLIV RLHGLLGNRW SLIAGRLPGR TDNEIKN
Stai_27949                         MGRAPCCDKA NVKKGPWSPE EDAQLKGYME KHGTGGNWIA LPHKAGLRRC GKSCRLRWLN YLRPNIKHGE FTDEEDRTIC SLFARIGSRW SIIAGQLPGR TDNDIKN
Stai_6424                          MGRSPCCSKV GLKRGPWSTK EDTLLTNFIH QNGEG-QWRS LPKKAGLLRC GKSCRLRWMN YLRPGIVRGN ITEDEEDLIV RLHGLLGNRW SLIAGRLPGR TDNEIKN
Stai_7654                          ANSGENVGEN TLKKGPWTSS EDDILIEYVT KHGEG-NWNA VRKYTGLARC GKSCRLRWAN HLRPDLKKEA FSPEEEYRII ELHAKMGNKW ARMAAELPGR TDNEIKN
Stas_10585                         MEKRAE---- -VKRGAWTKE EDTLLRICID KFGEG-KWHK VPIRAGLNRC RKSCRLRWMN YLRPNIKRGY FTKDEVDLIQ RLHKLLGNRW SLIAGRLPGR TANDVKN
Stas_1129                          MGRAPCCDKT KVKRGPWSPE EDNILKNYVQ QHGTSGSWIA LPLKAGLRRC GKSCRLRWLN YLRPNIKHGG FTEEEDNIIL TLYHNIGTRW SVIASLLPGR TDNDIKN
Stas_12182                         MGRSPCCSKV GLKRGPWSTK EDTLLANYIH QHGEG-QWRS LPKQAGLLRC GKSCRLRWMN YLRPGIKRGN ISEDEEDLIV RLHGLLGNRW SLIAGRLPGR TDNEIKN
Stas_14132                         MGRQPCCDKV GLKKGPWTAE EDKKLINFIL TNGQC-CWRA VPKLAGLLRC GKSCRLRWTN YLRPDVKRGL LSEYEEKMVI DLHAQLGNRW SKIASHLPGR TDNEIKN
Stas_4055                          MGRSPCCSKV GLKRGPWSTK EDTLLTNFIH QNGEG-QWRS LPKKAGLLRC GKSCRLRWMN YLRPGIVRGN ITEDEEDLIV RLHGLLGNRW SLIAGRLPGR TDNEIKN
Stas_9284                          ANSGENVGEN TLKKGPWTSS EDDILIEYVT KHGEG-NWNA VRKHTGLARC GKSCRLRWAN HLRPDLKKEA FSPEEEYRII ELHAKMGNKW ARMAAELPGR TDNEIKN
```

Figure S2. Amino acid alignments of the conserved region of the R2R3 Myb genes used for the NJ tree reconstruction in Figure 1. Amtri: *Amborella trichopoda*; At: *Arabidopsis thaliana*; Os: *Oryza sativa*; Sbai: *Scutellaria baicalensis*; Sind: *Scutellaria indica*; Sly: *Solanum lycopersicum*; Smon: *Scutellaria montana*; Spla: *Scutellaria playfairii*; Stai: *Scutellaria taiwanensis*; Stas: *Scutellaria tashiroi*.
